# Attitudes Towards the Donation of Human Embryos for Stem Cell Research Among Chinese IVF Patients and Students

**DOI:** 10.1007/s11673-018-9862-9

**Published:** 2018-07-02

**Authors:** Achim Rosemann, Huiyu Luo

**Affiliations:** 1 Department of Sociology, Philosophy and Anthropology, University of Exeter, Byrne House, FS3, Exeter, EX4 4PJ UK; 2 Centre for Bionetworking, School of Global Studies, University of Sussex, Brighton, UK; 3 Teaching Department of the Social Sciences, Xinxiang Medical University, Zip code, Xinxiang, 453000 Henan Province China

**Keywords:** Embryo donation, IVF stem cell interface, hESCR, human germ line editing, China

## Abstract

Bioethical debates on the use of human embryos and oocytes for stem cell research have often been criticized for the lack of empirical insights into the perceptions and experiences of the women and couples who are asked to donate these tissues in the IVF clinic. Empirical studies that have investigated the attitudes of IVF patients and citizens on the (potential) donation of their embryos and oocytes have been scarce and have focused predominantly on the situation in Europe and Australia. This article examines the viewpoints on the donation of embryos for stem cell research among IVF patients and students in China. Research into the perceptions of patients is based on in-depth interviews with IVF patients and IVF clinicians. Research into the attitudes of students is based on a quantitative survey study (n=427). The empirical findings in this paper indicate that perceptions of the donation of human embryos for stem cell research in China are far more diverse and complex than has commonly been suggested. Claims that ethical concerns regarding the donation and use of embryos and oocytes for stem cell research are typical for Western societies but absent in China cannot be upheld. The article shows that research into the situated perceptions and cultural specificities of human tissue donation can play a crucial role in the deconstruction of politicized bioethical argumentation and the (often ill-informed) assumptions about “others” that underlie socio-ethical debates on the moral dilemmas of technology developments in the life sciences.

## Introduction

The donation and use of human embryos and oocytes for research purposes has, over the last twenty years, sparked off widespread ethical debates at a global scale. Many of these controversies have focused on the use of these tissues for human embryonic stem cell (hESC) research, which involves the destruction of human embryos and the redirecting of their biological potential for scientific, medical, and commercial purposes. Since 2007, however, with the invention of induced pluripotent stem (iPS) cells, which allows the creation of pluripotent cells in an ethically less controversial way, debates regarding hESC have become gradually less pronounced. A widespread belief at that time was that fewer and fewer human oocytes and embryos would be needed and that the use of iPS cells (which can be generated from human somatic cells) would gradually replace the significance of hESC. In the last ten years, though, the use of hESC has remained important, which is reflected in various clinical trials and a continuing stream of publications that present findings from basic and preclinical research. Moreover, with the creation of the first hESC line from somatic cell nuclear transfer (SCNT) in 2013 (Tachibana et al. 2013), and more recently the use of human embryos for human germ line editing (Liang et al. 2015), the demand for human oocytes and embryos has once again increased.

An important aspect of the public and political controversies that have surrounded the use of human reproductive tissues for research, is that these debates have focused primarily on the moral, ontological, and religious status of human oocytes and embryos. They have mostly ignored the perceptions and crucial role played by women and couples in bringing these reproductive tissues into existence (Dickenson 2006) and the ways in which they are embedded in the wider social network of the family and community (Waldby 2008). Women and couples undergoing IVF treatment are the key providers of the “biological raw materials” that enable these forms of research and potential clinical applications. Sarah Franklin has in this respect introduced the term “IVF–Stem Cell Interface,” because the IVF clinic is the nexus that connects the intimate life worlds of IVF patients with the interests of stem cell researchers and the wider bio-economy (Franklin 2006). In contrast to the donation of sperm, the induced maturation and removal of oocytes in the IVF clinic is a risky process that is demanding from both a physical and an emotional perspective. It involves long-drawn-out regimes of medical examination and drug administering and finishes in a dicey surgical procedure. It is this demanding process of “women’s reproductive labor” (Cooper and Waldby 2014) that forms the vital core of an evolving biological economy which lies at the heart of the medical, scientific, and commercial benefits that the use of donated human reproductive tissues enables.

This under-communication of the role of women in public debates and of the bodily labour involved in hESC, SCNT, and germ line editing research, has given rise to questions about reciprocity, justice, and exploitation, with regard to the ways in which these tissues are sourced, circulated, and banked, and transformed into medical, scientific, and capital value (Franklin 2006; Parry 2005; Sleeboom-Faulkner 2014). One of the key questions that has emerged is whether there exists a balance between the value these tissues gain and the value they have for the persons who donate them and also whether there should be any form of payment or financial compensation for donors (Cooper and Waldby 2014).

Empirical research that has focused on the attitudes and actual experiences of embryo and oocyte donors for stem cell research has been rare. Virtually all of these studies have focused on the situation in Western societies, in particular the United Kingdom (Haimes et al. 2008), Denmark (Svendsen and Koch 2008), Switzerland (Scully et al. 2012), Germany (Krones et al. 2006), and Australia (Waldby and Carroll 2012). Empirical studies that examine the views of embryo or oocyte donors regarding stem cell research outside of Western Europe, the United States, and Australia have been extremely scarce. Research has been conducted on the sociocultural meanings of embryos among IVF patients in Japan (Kato and Sleeboom-Faulkner 2011), India (Gupta 2011), and China (Mitzkat, Haimes and Rehmann-Sutter 2010; Jin et al. 2013).

## Focus of Article and Key Findings

This article focuses on the attitudes, perceptions, and experiences of IVF patients in China regarding embryo donation for stem cell research. The paper also presents findings from a survey on the donation of human embryos for stem cell research that was carried out among Chinese students. There are two existing studies on embryo donation in Chinese IVF clinics. The first study, published by Chinese IVF clinicians (Jin et al. 2013), showed that the willingness among a sample of 386 IVF patients to donate embryos for stem cell research was low. The study cited lack of information on research purposes and distrust in science as the most important reasons for patients’ refusal. The second paper, by Mitzkat, Haimes, and Rehmann-Sutter (2010), which was based on a small-scale pilot study with five IVF patients, indicated that there was a widespread lack of understanding among women who were asked to donate their embryos for research. This study raised important questions about the handling of informed consent procedures.

In contrast to these two studies, this article engages in greater depth with the sociocultural frameworks that underpin processes of embryo donation in China. It also examines the ways in which the situated perceptions and attitudes of embryo donors are at odds with both bioethical discourse in China and the commentary of international observers. The paper shows that empirical evidence can play a crucial role in deconstructing politicized forms of bioethical argumentation and the (often speculative) assumptions about “others” that inform socio-ethical debates on the impact and moral dilemmas of technology developments in the life sciences.

The article illustrates that the attitudes and values on the donation of human oocytes and embryos in China are more diverse than has commonly been suggested. Chinese bioethicists, scientists, and regulators have repeatedly claimed that the donation of human oocytes and embryos does not constitute a problem for IVF patients in China. The bioethicist Renzong Qiu, for example, has argued that as a result of Confucian views many people in China presume that a person comes into being only at the moment of birth (Qiu 2007). This view has also been reiterated in interviews with Chinese stem cell scientists (Mann 2003; Sleeboom-Faulkner 2010). Another regularly expressed assumption among researchers in China is that because the country’s socialist government has for decades promoted atheism, religious concerns are by and large absent in Chinese society (Sleeboom-Faulkner 2010). The stem cell scientist Yang Xiangzhong has suggested that for these reasons “China has a cultural environment with fewer moral objections to the use of embryonic stem cells than many Western countries” (Yang 2004). Another widely held view is that unborn forms of human life in China are generally valued low because of the country’s family planning policies and the high number of abortions carried out during the last three decades (Cookson 2005). As pointed out by the demographer Yaqiang Qi: “abortion, which destroys embryos [and fetuses], has never been seen as wrong in Chinese society” (Qi, quoted in Mann 2003). While this statement is inaccurate from a historical perspective, as we will show in this paper, it is true that many commentators have assumed that China’s birth politics has created a cultural environment in which unborn forms of human life are generally devalued and dehumanized and that therefore the procurement and use of human embryos and fetuses for research is unproblematic (Mann 2003; Cookson 2005; Klein 2010). But China’s birth policy is also seen as conducive to the procuring of embryos for another reason: because the policy does not allow IVF patients to have more than one baby (since 2015, two babies), leftover embryos can no longer be used for pregnancy, and this increases the likelihood of their donation for research (Klein 2010).

While it is difficult to say in which ways exactly the sociocultural impact of the population policy has influenced attitudes and donation practices of human embryos in China, it is safe to argue—in the light of the empirical data we present in this article— that the above views and arguments are simplistic and often misleading. The findings from our survey and the in-depth interviews among IVF patients indicate that oocytes and embryos that are created in the context of IVF are entangled in a rich web of meanings, conceptions of value, values, emotions, concerns and social relations. This makes the question to donate these tissues for research often a difficult and highly personal decision. In the light of these findings, it is not surprising that a predominant number of IVF patients in China refuse to donate their spare embryos and oocytes for research.

The empirical sections of this article are structured in three parts. In part one, we conduct a brief literature review that introduces the key characteristics of valuation of early life forms in China. In part two, we present the findings from our survey study that was conducted among Chinese students. In part three, we present the data from our interviews with IVF patients, as well as findings from interviews with IVF clinicians and stem cell researchers. The article concludes that there is a need for more in-depth research into practices surrounding donation of human gametes, zygotes, embryos, and other body tissues in both developing countries and scientifically more advanced countries. Donation practices and corresponding regulations must be informed by these situated perspectives and the moral and social dilemmas that underlie the donation, use, and commercialization of human oocytes and embryos.

## Method

The data presented in this article are based on two inter-related studies that examined the attitudes towards the donation of human embryos for stem cell research in China. The first study involved in-depth interviews with fifteen IVF patients (twelve female and three male) and fifteen IVF clinicians from three IVF clinics in Southeast and Central China. The data were collected in February and March 2008. The interviews with IVF patients were conducted together with a translator who helped when needed. The interviews were recorded, and informed consent was obtained to use excerpts in social science publications. The quotations that are presented in this text are based on the transcription and translation of the audio recordings. The second study is a quantitative survey that was conducted in March 2008 of 427 students from two large universities in Wuhan, Central China. Of these, 250 respondents were female and 177 were male. Four hundred and nine students were completing an undergraduate degree (237 female and 172 male), and eighteen students were completing a postgraduate (master’s) degree (thirteen female and five male). Aside from gaining a general overview of our respondents’ attitudes on embryo donation for hESC research, we aimed to examine whether there were differences between male and female students as well as between medicine and non-medicine students. Our assumption was that medicine students had more knowledge of the characteristics and possible benefits of human embryonic stem cell research and that for this reason their attitudes might be more supportive compared to students from a non-medical study background. For this reason, we distributed our survey among students in the medical faculties of the two universities in which the survey was conducted and among students from a varied (non-medical) study background. Altogether, 227 respondents were enrolled in a medical degree programme (165 female and 92 male) and 170 students were enrolled in a (non-medical) study programme (85 female and 85 male). Respondents from non-medical disciplinary backgrounds included students from the natural sciences, humanities, social sciences, and business studies. Because the sample sizes of these divergent student groups were too small for systematic comparisons, we collapsed all non-medical students into a single category and solely compared the views of medical students with the views of non-medical students. We distributed our questionnaires to students in lecture halls at the end of lectures after obtaining permission from professors and teaching staff and in the reading rooms of the two universities’ main libraries.

We have selected university students for a variety of reasons. First, access to students is easier to negotiate than a broader segment of the public, which would have required permissions from multiple institutions and work units. Second, the financial budget for this research was limited. For monetary and organizational reasons, a survey among a more representative subset of the Chinese population was not possible. Third, students can be expected to have a sufficient knowledge basis to answer the questionnaire. This is not necessarily the case for other segments of China’s population. Especially, older people and persons with a low educational background would possibly have struggled with the questionnaire. Another reason is that students are the future leaders and workforce of contemporary societies, whose lives and reproductive decisions are likely to be affected by the availability of artificial reproductive technologies and regenerative medicine. Their ideas and attitudes are relevant to ethicists, policymakers, and academic discourse.

Clearly, university students are not typical of the public of large. Moreover, because the survey includes data from a relatively small set of students, from only two universities in one city, representativeness of the student population in China as a whole is limited. Generalization to a larger sample of students or the wider public must be made with caution. Another limitation of this study is that students are asked to consider a hypothetical situation, but their way of thinking when answering the survey does not necessarily correspond with their actions and reasoning were that situation to really happen. The validity of the survey data in relation to actual practices may therefore be low. To mitigate this problem, we present in this article first of all the findings from the survey and then continue with the findings from our qualitative study, where we present the views of IVF patients towards embryo donation (where the option to donate embryos was no longer hypothetical but very real). The survey has been co-designed by the first and second author of this paper. The survey explained the purposes of and prospects for the use of IVF embryos for human embryonic stem cell (hESC) research in written form on the first page of the survey form. This introduction also stated that donated embryos would be destroyed and would no longer be used for reproductive purposes. The survey questions explored attitudes regarding the use of embryos and oocytes for stem cell research, as well as corresponding values and beliefs. The questionnaire included multiple-choice and open-ended questions to which respondents were asked to provide handwritten comments. Two hundred and twenty survey participants provided handwritten comments and explanations of their viewpoints. These comments were translated from Mandarin Chinese to English by translators in China. Data analysis involved simple descriptive statistics and was carried out with SPSS 22.

## Embryo and Oocyte Donation for Stem Cell Research in China

The procurement of human embryos for stem cell research in China is regulated in line with international standards adopted in most high-income countries. Chinese regulatory guidelines require that IVF clinics set up ethics committees and that these committees approve the donation of human embryos for research. Embryo donation must be voluntary and be based on informed consent. Hormonal super-stimulation to increase the harvest of oocytes during an IVF cycle is forbidden. Also, in contrast to the United States, the buying or selling of human oocytes is banned. Moreover, as in most countries, human embryos cannot be used for the derivation of hESC after fourteen days post-conception. Violations of these rules can be legally prosecuted, and IVF clinics and individual doctors can lose their licenses (Warrell 2009). Also, with regard to the donation and use of human embryos for human germ line editing research, as Zhai, Ng, and Lie (2016) have pointed out, no major regulatory differences between China and other scientifically advanced countries exist. Genetic modification of human gametes, zygotes, and embryos for reproductive purposes is prohibited but can be allowed for research purposes under certain conditions. Despite these similarities at the level of formal regulatory frameworks, there are several contextual factors that are specific to China and which are likely to influence the attitudes, perceptions, and experiences of Chinese embryo donors. The first factor is that there have been problems with the enforcement of regulatory frameworks in other areas of the biosciences. In clinical stem cell research, for instance, problems with the implementation of regulatory provisions have resulted in uncontrolled interventions that have been offered on a for-profit basis (Sui and Sleeboom-Faulkner 2015). Also, regarding the implementation of hESC research, various challenges have been reported. Zhai (2007), for instance, has mentioned that, due to the absence of a registration and licensing infrastructure for researchers that conduct hESC research, consistent controls of ethical standards for hESC research are difficult to realize. The enforcement of reliable controls is complicated especially because of the large territory of China and the high number of research institutions. The second factor concerns the changing role of religion in Chinese society. Under communist rule, in particular in the Maoist period but also during the post-reform era, religious practices and beliefs have often been actively suppressed (Potter 2003). As reported by Sleeboom-Faulkner and Patra (2008), due to the (assumed) secular nature of Chinese society, it was repeatedly claimed that the religious scruples that characterized public and political debates in the United States and many European societies would not exist in China. In recent years, however, religious controls have partially loosened, and a variety of different religious traditions have been revived or newly introduced into Chinese society (Marsh 2011). Religious perceptions, as well as values and norms from more “traditional” folk beliefs, are thus likely to play a more important role for embryos donors than is commonly suggested. The third factor is that Chinese citizens have experienced three decades of population policy, in which the female body has been the locus of state-directed reproductive control and intervention. However, with regard to the sourcing of human embryos and gametes, concerns that similar forms of state-induced pressure might be exerted on women or IVF couples (Cookson 2005) have clearly proven wrong (Sleeboom-Faulkner 2014). Nevertheless, there is reason to believe that China’s population policy is influencing women’s and couple’s perceptions of the value of their oocytes and embryos. Mitzkat, Haimes, and Rehmann-Sutter (2010) have suggested, that due to the one-child policy the practical significance of “spare” embryos disappears once a baby has been born and that this may increase the willingness to donate embryos for research. However, with the recent change of China’s population policy from a one-child to a two-child policy, the legal situation for IVF patients has once again changed. Thousands of (frozen) embryos have suddenly become legally available for IVF couples to have a second child (Wahlberg 2016).

## Bioethical Perspectives on Unborn Human Life in China

There is no cultural consensus or single bioethical view on the use, donation, and destruction of human embryos and fetuses in China. With regard to the use of embryos for human embryonic stem cell (hESC) research, bioethicists in China have, since the early 2000s, called for reliable standards and the adoption of the bioethical principles of autonomy and informed consent, including the right to refuse embryo, oocyte, and sperm donation for research and the recognition that IVF patients are the legal owners of their embryos and gametes (Doering 2004; Cheng et al. 2006). However, divergent viewpoints exist with regard to the ways in which the perspectives of IVF patients (as potential donors for stem cell research) have been represented and problematized.

### Confucian-Based Interpretations

The influential bioethicist Renzong Qiu, for example, has stated that due to its status as a potential human being the embryo deserves due respect and that the interests and position of possible embryo donors must be well protected by regulatory safeguards (2007). However, according to Qiu there are fewer moral obstacles to the donation of human embryos for research in China than in Western and especially Christian societies (2007), where the embryo is often already seen as the carrier of a soul or spirit (Walters 2004). A central reason Qiu cites for this is the cultural influence of Confucian moral philosophy. The ethicist Yanguang Wang, a colleague of Qiu, summarized his position as follows (Wang 2003):

According to the accepted Confucian view, a person begins with birth. A person is an entity that has a body or shape and psyche, and has rational, emotional and social–relational capacity. So a human embryo is not a person, a personal life. Destroying an embryo as well as an abortion should not be taken as killing a person. However, a human embryo is a human biological life, not merely stuff, like a placenta. So it deserves due respect. If there is no sufficient reason, it won’t be permissive to manipulate or destroy it. Saving a great number of human personal lives can be a sufficient reason.

Hence, for Qiu, in line with Confucian reasoning, a fetus acquires personhood only with birth. The unborn embryo or fetus is not yet a person or a personal life, and its use for research is justified, provided there is sufficient reason. The therapeutic potential of hESC research is seen to be such a reason. Another explanation of Confucian notions of personhood has been provided by the ethicist Edwin Hui (2003). According to Hui, from a Confucian perspective, personhood is acquired gradually through social practice. It is thus not an innate or given property but develops over time through socialization and an individual’s social relations with the family and society (Hui 2003; see also Klein 2010). According to Qiu, this view has important implications for bioethical interpretations of the beginning of life and the value of unborn life. If being a person begins at birth, and personhood is acquired gradually through social behaviour, the value of unborn human life, especially at its initial stages, is low and less worthy of protection. It is a form of human biological life but not yet a person, and the destruction of a human embryo should not be taken as killing a life (Qiu 2004). According to Qiu, this is a widely held view in China and a central reason why IVF patients (in their role as potential embryo donors) can be expected to have less moral scruples or objections to the donation and use of their embryos for stem cell research (Qiu 2007; *cf*. Klein 2010). Yali Cong, a bioethicist from Peking University’s Health Science Centre and a former student of Qiu, reaches a similar conclusion. Cong (2008, 23–24) argues that:

[From] the mainstream of Chinese culture, that is, the tradition of Confucianism, the life of a person can be divided into two aspects: biological life and social life. Biological life does not have an inner character of “sacredness”, as the classical Confucian philosopher Xun Zi argued: the reason a person can be treated as a person is because he has *yi* (righteousness); otherwise, there is no difference between people and animals. […] The idea of social and moral life is so strong that people don’t take human biological life too seriously, let alone the embryo, or even the infant just after birth.

A related point is that, according to Cong, from the perspective of many people in China, “life comes from the parents, not from God.” As she points out:

This is the main reason individual persons, especially children, do not have a great deal of room for independence. Accordingly, parents have the right to make decisions for their children, even the right to decide about life. Thus, there is no obstacle for a woman who wants to stop her pregnancy, especially within the first three months. (Cong 2008, 25).

A practical problem with this view, and also with the arguments of Qiu and Hui above, is that these statements are primarily based on philosophical reasoning rather than systematic social science enquiries among Chinese citizens. As also stated above, a survey study by a team of IVF clinicians amongst almost four hundred IVF patients found that more than half (58.8 per cent) of all respondents indicated that they would refuse donation of their embryos for hESC research. A group around the Shanghai-based bioethicist and policymaker Chingli Hu arrived at similar conclusions. Based on interviews with religious and women’s organizations in China, this team concluded that a significant group of interviewees expressed moral concerns regarding the use of IVF embryos for hESC research (Hu 2009). These findings cast doubts on the Confucian-based argument that people in China generally consider the value of unborn human life low, and they indicate the need for more systematic empirical research, especially regarding the perceptions of women and couples undergoing IVF.

### Bioethical Views on Abortion and the One-Child Policy

Another crucial issue in identifying the characteristics of the valuation of unborn human life forms in China is the extent to which, and the ways in which, perceptions of the use of embryos for hESC have been influenced by the legislation, discourse, and the large number of abortions that have been conducted under China’s population policy. As stated above, various authors (Mann 2003; Cookson 2005; Klein 2010) have suggested that the cultural environment created by China’s restrictive birth politics has significantly influenced public attitudes and ideas on the procuration and use of embryos for hESC research.

Bioethical positions in China have generally endorsed the population policy. In line with the policy’s rationale, the large number of abortions that were conducted in the context of the policy were seen as justified for the common good, as overpopulation and rapid population growth in China threatened the whole society (Jiang and Liu 2016). Nonetheless, bioethicists such as Renzong Qiu have repeatedly pointed to some of the problematic aspects of the one-child policy, such as sex-selective abortion and the lack of systematic controls on policy implementation at the level of villages and towns (Nie 2011). Despite the widespread support for abortion under the one-child policy, it would be misleading to assume, as the demographer Yaqiang Qi (referred to already above) has claimed, that “abortion […] has never been seen as wrong in Chinese society” (Qi quoted in Mann 2003). This is not true. During both the late Qing Dynasty (from *c*. 1880–1911) and the Republican years of China (1912–1947) abortion was prohibited, criminalized, and in some provinces equated with killing (Long 2012). The eminent ethical scholar of the Republican period, Guobing Song, suggested in 1933 that “the human fetus from the moment of its formation has its life and human rights, and the entitlement to be protected” [tai’er zi jie tai yihou, ji you qi shengming yu renquan, qie you qi baozhang shengming zhi quanli] (Song 1933, in Long 2012, 98).

While abortions were still carried out during this time, most abortions were conducted secretly, and physicians worked under the risk of being criminally convicted (Long 2012). Abortion only became legal after the foundation of the People’s Republic of China in 1949 when they were allowed as a part of family planning on a voluntary basis and since the 1980s as a central element of the country’s one-child policy (1980–2015). According to China’s Ministry of Health, reportedly 336 million abortions have been conducted in China since 1971, the majority on account of the one-child policy (Moore 2013). Since the early 2000s, an increasingly critical body of bioethical literature has emerged on China’s birth politics. These studies have pointed in particular to the individual and social suffering caused by the policy. This is best epitomized by the bioethicist and social scientist Jingbao Nie’s study *Behind the Silence.* As Nie states: “the one-child policy and the application of the authoritarian model have instead caused massive suffering to Chinese people, especially women, and made them victims to state violence” (Nie 2005). The awareness of the human cost of the policy, together with the reduction of China’s birth rate and a rapidly ageing society, have resulted in the gradual softening of the policy in recent years and in the transition towards a two-child policy since January 1, 2016 (Tian 2015). In legislation, unborn human life (up to the later stages of fetal development) was primarily seen as a biological entity that could be separated and destroyed without much ethical consideration. In legal terms, the ownership of unborn “surplus babies” (unborn babies of families that had already one child) was transferred from the parents to the state, which had the authority to enforce abortion and to execute sanctions if people resisted the policy.

As developments in other jurisdictional domains in China show, however, this “cold” or “pragmatic” view on unborn life forms has not been automatically transferred to other policy areas. In Chinese patent law, for instance, a completely different ethical discourse on the valuation of prenatal human life forms has recently emerged (Jiang 2016). As Article 9.1.1.2 in Part Two of the 2010 Chinese Guidelines for Patent Examination state: “The human body at various stages of its formation and development, including a germ cell, an oosperm, and embryo and an entire human body, shall not be granted the patent right.”^1^ This prohibition to patent unborn human life or other parts and tissues of human bodies is explained by a morality clause (in the above guidelines), which states that: “when the commercial or industrial use of an invention is unacceptable to the public and not recognized by common moral standards, a patent right cannot be granted” (Jiang 2016).

Hence, in contrast to the “cold” logic of dispossession of the population policy (which legitimizes individual suffering by promoting the interests of society as a whole), patent law, and also the law for artificial reproductive technologies (ART law), and the regulations for hESC research––all emphasize the protection of interests and rights of individual citizens.

In the context of the donation and transfer of human embryos and gametes for reproductive purposes (as defined in ART law) and for research purposes (as defined in the hESC regulation) both the biological originators and the embryos (and gametes) themselves are seen as requiring special protection and are granted rights that prevent unauthorized removal, irresponsible use, and commodification.

In light of this conflicting and changing legal situation, it is extremely difficult to assess the impact of China’s population policy on the valuation of embryonic life forms in the context of embryo donation for hESC research. While we can assume that thirty-five years of population control have left a mark, one has to be careful not to overestimate the influence of the population policy or to confuse official policy positions and discourse with the opinions and perceptions of ordinary people, physicians, and scientists. As our empirical findings in the next sections show, the perceptions and ascribed value of human embryos are diversified and complex. For many of our respondents, the donation of human embryos for research or commercial purposes seems unthinkable. It seems misleading and wrong to assume that—as a result of the impact of the one-child policy—human embryos are generally seen as being of low value in Chinese society.

Our empirical data suggest that a conflation of the moral positions embedded in the population policy (in which prenatal human life has been portrayed as mere “biological matter” that could be disowned and destroyed without many ethical concerns) with the attitudes and perceptions of ordinary people is misleading. As the findings from our study suggest, forms of embryonic life in China are entangled in a rich web of overlapping and sometimes contradictory layers of meaning, values, emotions, and social relations, of which analysts, policymakers, researchers, and clinical staff should well be aware.

While our survey data and other studies (Jin et al. 2013; Mitzkat, Haimes and Rehmann-Sutter 2010) indicate that this is likely the case for a large proportion of the general public, this seems especially true for IVF patients, for whom embryos and gametes are of particular significance because they embody the hope for a child after a lengthy and often painful period of infertility. Indeed, it is a significant shortcoming that bioethics discourses in China (and many other countries) have for a long time approached the moral questions and perceptions of potential embryo donors as a more abstract problem, which has been discussed in terms of broader moral and cultural categories and assumptions, rather than with regard to the specific context of IVF and the perceptions of IVF patients. As this article and many others (Haimes 2008; Scully, Rehmann-Sutter, and Porz 2010) have shown, the valuation of human embryos and gametes has very particular characteristics in the IVF clinic. The context of infertility and hope, and also the technical process of IVF—in which human embryos are produced outside the human body, become visible through imaging technology, and are assessed in terms of their reproductive quality—create specific forms of valuation, emotions, and affective bonds that differ from a “normal” pregnancy and that remain unaccounted for in the context of a hypothetical evaluation of embryo donation by non-IVF patients.

## Findings from the Survey among Students

In this section we will present data from the survey. This includes both quantitative and qualitative data, the latter in the form of handwritten comments through which survey participants could further explain their viewpoints. Among the 427 students who returned their questionnaire, only 48.7 per cent indicated that they would agree to the donation of their embryos for hESC research. Interestingly, among female respondents the willingness to donate embryos was significantly higher (52.2 per cent) than among male respondents (44.1 per cent). Among medical students, on the other hand, the readiness to donate (49.4 per cent) was only a little higher than among non-medical students (47.6 per cent). Of the total number of respondents, 50.8 per cent (48.0 per cent female vs. 54.8 per cent male) said they would refuse donation and 0.5 per cent remained undecided. The number of refusers among medical students was only slightly lower (49.8 per cent) than among non-medical students (52.4 per cent). It is worthwhile to note that the percentage of people who refuse to donate their embryos for hESC research in our survey is lower than the results from Jin and colleagues’ study on the views of 363 Chinese IVF couples on the donation of their frozen “spare” embryos (Jin et al. 2013). According to these authors, 58.8 per cent of all couples preferred to discard surplus embryos instead of donating them to research. However, the percentage of refusers in our survey is higher than the results from a survey study in the United Kingdom, where 46 per cent of respondents opted against donation (Scully et al. 2012).

Among the 50.8 per cent (n=217) of the survey’s respondents who refused donation, the most widely supported reason was “I do not donate, because using an embryo is the same as taking a life.” Of this subgroup, 57.1 per cent supported this statement. This statement found more support among male respondents (61.9 per cent) than among female respondents (53.3 per cent). Among medical students, support for this claim (57.8 per cent) was roughly the same as among non-medical students (56.2 per cent). This is a surprisingly high percentage, which echoes one of the key complaints against the use of embryos for hESC research in many Western societies.

While opposition to the use of human embryos is unlikely to be as pronounced as among the religious right in the United States, this is nonetheless an interesting finding because it runs counter to most bioethical claims in China. It also conflicts with assumptions regarding the cultural impact of the one-child policy (Mann 2003; Cookson 2005). The issue was qualified in several of the survey respondents’ handwritten comments:

To donate an embryo to research is equal to killing a life. I think life cannot be destroyed casually. (Student, medicine, female, 25 years)

The embryo is the descendant of me and my wife. It is an organism and it can’t be killed. (Student, financial engineering, male, 23 years)

A possible reason for the extensive support for this statement might be that more than half of all survey respondents (n=427) indicated that in their view the life of a human being starts at the initial stages of embryogenesis. This stands in sharp contrast to the (assumed) Confucian-based perception of embryo donors in China, according to which a large proportion of people (reportedly) think that human life starts only at the moment of birth (Qiu 2007; Cong 2008).

In our survey, 51.1 per cent of all respondents replied to the question “when do you think the life of a human being starts?” by selecting the option “at the moment of fertilization.” This view was shared among 50.8 per cent of all female respondents and among 51.4 per cent of all male respondents. The number of medical students who endorsed this idea was 54.1 per cent, higher than among non-medical students, of whom only 46.1 per cent endorsed this statement. Another 37.2 per cent of the total number of respondents saw the starting point of a human life at the moment “when a fertilized egg cell has evolved to an embryo.” A higher proportion of female respondents shared this view (40.8 per cent) compared to male respondents (32.2 per cent). The number of medical students who supported this statement (35.8 per cent) was slightly lower than among non-medical students (39.4 per cent).

Altogether only 10.5 per cent of all students considered the starting point of human life to be situated at a later moment during gestation: 2.6 per cent opted for “the development of the nervous system” (2.0 per cent of all female respondents vs 3.4 per cent of male respondents; and 2.7 per cent of all medical students vs 2.6 per cent among non-medical students). Of all respondents, 2.3 per cent argued in favour of “the development of the organs” (2.8 per cent of female respondents vs 1.7 per cent of male respondents; and 3.1 per cent of all medical students vs 1.2 per cent of non-medical students). Remarkably, only 5.6 per cent of all respondents supported the Confucian perspective that “the life of a human being starts at the moment of birth.” Of interest is that male respondents supported this idea to a much higher degree (10.2 per cent) than female respondents (2.4 per cent). Also, support among medical and non-medical students to this statement was significantly different. While 9.4 per cent of all non-medical students endorsed the view that the life of a human being starts only at the moment of birth, the idea was supported only by 3.1 per cent of all medical students. These findings suggest that medical knowledge on embryogenesis, as well as gendered perspectives, play a significant role in supporting the idea that human life starts only at birth. While altogether 94.4 per cent of all respondents imagine the starting point of human life to be at an earlier (prenatal) stage, it is nevertheless remarkable that more than 10 per cent of all male respondents supported the idea that life starts at birth. One way to explain why this view was only endorsed by 2.4 per cent of all female respondents is that the embodied perspective (or imagination) of pregnancy instils an earlier and closer affective bond towards an unborn child than is the case with men (and especially the very young group of male respondents to this survey). These findings suggest that the view that human life starts only at birth is much less common in Chinese society than is often claimed. They also suggest that gendered perception and the experience of pregnancy, as well as exposure to more detailed knowledge on what happens during embryogenesis, are important factors in the emerging of other viewpoints (at which the starting point of human life is imagined to be at an earlier stage). More research into what respondents exactly mean when they say that a human life starts at birth, or at any other point, would be required to interpret these data in a way that makes them relevant for policy decisions. Nevertheless, the fact that more than 90 per cent of students located the starting point of life at a much earlier stage than that which is commonly stated as the normal Chinese bioethical view is remarkable and requires further investigation.

Another statement that was widely supported among respondents who refused to donate (n=217; 58 per cent of all respondents) was: “I do not donate my embryo, because I am afraid of emotional or psychological consequences” (35.9 per cent). This view was shared among 37.5 per cent of female respondents and 34.0 per cent of male respondents. Among medical students who refused donation, this statement was endorsed by 36.7 per cent, compared to 34.8 per cent of non-medical students who indicated they would reject embryo donation. Such fears were also reflected in several of the handwritten comments:

It [embryo donation] may have consequences for people in a spiritual and psychological sense. Also, it may bring conflicts with morals and ethics. (Student, Chinese literature, female, 23 years)

It may mentally hurt the person who donates. (Student, computer science, male, 21 years)

“I do not donate my embryo, because my parents will probably disagree” was another relatively widely supported reason to refuse donation. Of all 217 respondents who had indicated they would refuse donation, 28.6 per cent endorsed this statement (50.8 per cent of all 427 survey respondents, as mentioned above). This concern was shared by 30 per cent of all female respondents and 26.8 per cent of all male respondents who said they would refuse embryo donation. Among medical students who would reject donation, support for this statement was more than 8.0 per cent higher than among non-medical students: 32.0 per cent among medical students versus 23.6 per cent of non-medical students.

A slightly higher number of the 217 respondents who had indicated they would refuse donation of their embryos said they would not donate because their partner would probably disagree. This statement was supported by 33.2 per cent of all respondents from this subset of respondents. Interestingly, the number of men who endorsed this statement (40.2 per cent) was much higher than among female participants (27.5 per cent). This indicates that many male respondents in this survey regarded the needs of their female partners as more important than their own views. Among the medical students who would refuse donation, endorsement of this statement (34.5 per cent) was more or less the same as among non-medical students (31.5 per cent ). As the following quotations reveal, numerous respondents would personally agree to donate their embryos but would refuse donation due to respect for (and perhaps also fear of) the opinions of parents, partners, and family members. Various respondents complained about “traditional,” “feudal,” or “conservative” opinions, and said they would actively try to persuade their parents and/or partner:

My ancestors and parents stick to their feudal thoughts. I’ll try hard to change their mind. Such donation can help others as well as ourselves. But if my parents object strongly, I’ll accept their opinion and give up. (Student, medicine, male, 20 years)

The embryo is the fruit of love between my husband and me. It is not only life but also the hope of the family. In its cells are our genes. So, my parents and forefathers will not agree. If after negotiating with my husband we decide to donate it for medical research, then I’ll try to persuade my parents and forefathers to accept this. (Student, life sciences, female, 23 years)

These statements reflect a weighing between “traditional” values on the one hand (as embodied by the attitudes of the parents and ancestors) and the logic and needs of “modern life” (as expressed in the desire for medical progress by many of the younger persons). Some of the students indicated they would simply ignore the opinion of their parents and donate their embryos to research:

My parents and ancestors are all conservative in thought, so they may not agree. But if I will insist on donating, I will stick to my principles. (Student, chemistry, male, 24 years)

I think tradition is the obstacle here. As traditional people, my parents will not agree. I’ll manage the donation plan myself. (Student, sociology, male, 22 years).

Another interesting finding of the survey was that 81.3 per cent of all respondents (n=427) supported the statement that “human beings have a soul (*linghun*) or spirit (*jingshen*).” This view was shared by a considerably higher number of female respondents (85.2 per cent) compared to male respondents (75.7 per cent ), as well as a higher proportion of medical students (84.4 per cent) than non-medical students (76.5 per cent ). The notion that “the human embryo has a soul or spirit” was supported by 45.2 per cent of all respondents. Again, female study participants endorsed this statement significantly more (50.4 per cent ) than their male counterparts (37.9 per cent), and also among medical students, support for this view was more than ten percentage points higher (49.8 per cent) than among non-medical students (38.2 per cent). Considering that 85.3 per cent (n=398) of all respondents indicated they have a non-religious background, these numbers were surprisingly high. Among respondents with a religious background, however, the support for the idea that embryos have a spirit/soul was even higher: 75.9 per cent.^2^ The idea that embryos have a spirit/soul was supported by 75.0 per cent of all female religious respondents and by 77.8 per cent of all male religious respondents. Among religious medical students, the support for this notion was 78.3 per cent and more than ten percentage points lower among religious non-medical students: 66.7 per cent. Further research would be required to explore how both “religious” and “non-religious” groups conceive of the notions of “soul” and “spirit,” and in which ways these conceptions influence donation decisions.

## The Perspectives of IVF Patients

This section explains the value and significance of in vitro fertilized embryos for women and couples undergoing IVF treatment. It shows that IVF embryos are closely entangled with the social, physical, and emotional perceptions of their biological originators and wider kinship groups. The section clarifies, furthermore, that value conceptions of embryos and attitudes regarding research use are intimately shaped by the experience of infertility, the emotional pressures that emerge in the context of the infertility treatment, and the specific conditions through which embryos are created, stored, and applied in the IVF clinic. Perceptions of IVF embryos and embryo donation can undergo important changes in relation to classifications of the embryos’ “quality,” the state and location of embryos (fresh in a Petri dish or cryopreserved in a freezer tank), and reproductive success (i.e. before and after a successful pregnancy).

### The Value of IVF Embryos in the Context of Infertility

Value conceptions of IVF embryos are influenced by the experience of infertility and related sociocultural pressures to give birth to a child. For persons undergoing infertility treatment, the in-vitro-generated embryo signifies a source of profound hope and value. It constitutes a form of “reproductive capital” that, after the experience and diagnosis of infertility, render a long-cherished but repeatedly discouraged dream back into the realm of the achievable:

We had waited long for a baby. At the beginning of our marriage already we started to discuss our baby’s name. But then I felt disappointed. Then when the embryos were created in the laboratory and stored in the [nitrogen] tank, I started to think—these embryos are my hope, my hope for a child, my hope for the future. (Female IVF patient, 30 years)

The IVF embryos form a scarce and precious substance that is embedded in a network of emotional, physical, and kinship relations. With their potential to reverse personal and collective suffering, they epitomize a pinnacle of hope for a better and happier future:

I have been worried for a period of five years now, and during this period I always felt stressed and anxious and kind of blue and depressed in my heart. Also, I felt not so energetic. But now, where I know that I have this tiny baby in my body, I feel everything is so bright, and the future is also bright and my parents and the whole family will be happy for me. (Female IVF patient, 29 years)

While it can be argued that such expressions of joy characterize the experience of (successful) IVF patients everywhere, in the sociocultural fabric of China such emotions are intensified by the following factor. First, in the patriarchal and patrilineal tradition of Chinese society, it has been a central moral obligation for sons to carry on the family line, an attitude that is influential also in the present (Greenhalgh and Winckler 2005). Moreover, as IVF patients and clinicians have repeatedly stressed in interviews, even today infertility is widely regarded as a form of personal failure. Infertility and childlessness, therefore, are often paralleled by feelings of shame and fear of stigma. Such anxieties exist in particular in rural areas, where infertility becomes a public issue sooner or later:

To have a child is like your destiny. If you have a baby, then that is your parents’ contribution, your parents’ achievement. But if you have no child, maybe the gods have punished you. Maybe the neighbourhood will blame the parents: “they must have done some bad things; that is why their children can have no baby.” (Mid-career IVF clinician 1)

Infertility is explained here from within the beliefs of folk cosmologies, which can give rise to painful accusations and stigmatization of the infertile couple and their parents. Whilst the case may be extreme, it shows that couples’ efforts to give birth to a child are underpinned by strong normative pressures. This situation is likely to have a significant impact on attitudes to the donation of IVF embryos.

### Differentiating Quality/Stratifying Value

Despite the significance of IVF embryos for patients, in the course of the infertility treatment the conceptions and feelings of IVF patients regarding their embryos are subjected to changes. In the IVF clinic, patients are exposed to new forms of expertise and explanations and their embryos are subjected to the rigorous testing of quality, morphology, and reproductive viability. In other words, during their treatments patients learn to think about the characteristics and value of their embryos through the technical categories and quality parameters of the IVF space. Most importantly, embryos are ranked through a classification system that defines their reproductive value on a continuum from “good” to “bad.” This process consigns IVF embryos to various destinies. Those of the highest quality group are usually transferred directly into the uterus (so-called “fresh embryo transfer” which offers the highest likelihood for a pregnancy). Often, however, more “high-quality” embryos are produced than can be transferred into the uterus at one time. In this case, these embryos are stored and frozen in tanks of liquid nitrogen for future use, together with the embryos of lower-quality groups. Embryos whose reproductive potentiality is judged low, unpredictable, or absent are classified as “waste.” This limits the future use of these embryos to two possibilities: disposal or (provided researchers are interested in these “low-quality” embryos) donation to the research lab. The fact that IVF embryos are frozen in tanks of liquid nitrogen constitutes a source of concern for many potential parents:

I am so afraid that the freezing will damage the quality of my embryos. You know, before freezing the quality of my embryos was very good—first grade. (Female IVF patient, 32 years)

I hope so much that these embryos have a good quality and that they can make it and I will have success. (IVF patient 31 years)

Hence, in the course of the IVF treatment, patients learn to think about their embryos within a new set of vocabulary and parameters. These factors play an important role in the shaping of attitudes toward embryo donation. The restructuring of ideas, attitudes, and mental images of patients’ embryos in terms of categories of reproductive viability clearly facilitates the attempts of clinicians or stem cell researchers to motivate IVF patients to donate their spare embryos for research. At the start of their treatment, IVF patients attribute equal value to their embryos. However, once the IVF embryos are produced and assessed in the clinics, some embryos are labelled as being of lower reproductive value than others. This stratification has an important effect. The privileging of “high-quality embryos” in the context of IVF is accompanied by the consistent devaluation of the category of the “poor-quality embryos,” which are then defined as “disposable” or “waste.” As Cussins has pointed out, this rhetoric of stratified value justifies the exemption of the “low-quality” embryo from “the moral and legal standards that apply to embryos as potential sources of life” (Cussins 1998, 186). Without this step, the removal of these tissues or their donation for research would be more difficult to legitimize. It should be noted, though, that from a biological perspective, the argumentative pillars on which this “exemption of moral and legal standards” is based are not entirely stable. As Chen et al. have shown through their work with declassified embryos, classifications of embryos as “waste” are never absolute indications. Some of these embryos still have a substantial reproductive potentiality and could result in a successful pregnancy. Because these embryos’ pregnancy rate is not as elevated, however, as that of the higher-graded embryos, their usage in the IVF clinic is banned (Chen et al. 2005). For hESC researchers, these “poor-quality” embryos can still form a valuable resource, an indication that reveals the ambivalence and multi-layeredness of value conceptions of embryos. It is important, in this respect, also that the stratified value conceptions of the IVF clinic can conflict with culturally transmitted beliefs and social norms, for which differences in quality are irrelevant. This will be discussed further below.

### The IVF Embryo as Emotional Object

The emerging of emotional bonds on the part of IVF patients toward their embryos is not a linear process and is experienced differently at different stages. Most of the women with whom I spoke said they would not build an emotional bond with embryos that were defined as “low quality.” None of these persons considered the discarding of these embryos as a problem, a situation that clearly facilitates the donation of these embryos for research.

I knew before that this situation will emerge … that there will be many embryos, and that not all can be used. I know this consequence, and I consider this as a part of the treatment. (Female IVF patient, 28 years)

Embryos that were defined as “high quality,” on the other hand, were typically much valued, and some women reported that strong affective ties could emerge toward their frozen embryos over time. During a first IVF cycle, when a couple’s “fresh embryos” were transplanted, emotional ties with the frozen embryos were reported to be low:

In my mind, these frozen embryos are only very tiny, tiny round things—little stuff there. Because it is not in my body I think it is just some cells. I cannot say that I have a special emotion to it. (Female IVF patient, 31 years)

In my body, the contact with the embryo is stronger and the emotion is stronger. (Female IVF patient, 30 years)

These testimonies suggest that emotional bonds toward IVF embryos build up in a gradual process. Due to the spatial distance and the uncertainty whether the frozen embryos will “survive” or “maintain their good quality,” women seem first of all to avoid building up a stronger emotional attachment to these tissues. However, this should not be misread as indifference. Rather, it may form a kind of self-protection. Virtually all patients experienced anxieties about these embryos, and almost unanimously they hoped that “they are kept safe there” and that the “doctors will take good care of them.” Our data show that, if the initial IVF cycle with “fresh embryos” was unsuccessful, the feelings and ideas of IVF patients toward their frozen embryos changed. A female patient told us that after her first unsuccessful IVF cycle, she started to imagine of her frozen embryos as “very little children” of which she hoped that “they have it good out there” and that they are “well protected so that they cannot be stolen and used for a pregnancy by another couple.” These findings indicate the likelihood of important changes in attitudes towards embryo donation as the IVF process proceeds.

Emotional bonds with the embryos in the nitrogen tanks can also last, or, as the following example shows, even intensify—once a pregnancy has been successfully initiated. These bonds can prevent the willingness of women to donate their embryos. The following excerpt is from a female IVF patient who—just before we met her—had heard that she had become pregnant:

I want to keep my [frozen] embryos for a long time. I really cannot consider giving them away now. Maybe later, when my child is four or five years old … but also after five years I would not like to give them away all. I still would like to keep some. (Female IVF patient, 29 years)

Perhaps because her IVF embryos had now proved their reproductive viability, this woman felt an intensive emotional attachment toward her frozen embryos. The donation of these embryos for research or disposal was, at least at the moment we spoke to her, considered unthinkable. Other women may feel less intense attachment and be more willing to donate their spare embryos for research. However, the idea that the frozen embryos should be kept for some years was widespread. The most frequent reason was that during or after pregnancy something might happen and that further embryos would be needed. The frozen embryos, from this perspective, constitute an important “reserve reproductive capital,” a source of value on which one can rely when really needed. Together, these findings show that detailed knowledge on the (changing) meanings and value conceptions of embryos during the IVF treatment is of high relevance to understanding the ways in which attitudes towards embryo donation are shaped in the context of IVF. This insight has not yet been fully explored in policy discussions about the use of embryos for research.

### The Embryo as Part of the Family and Kinship Group

A final aspect that should be highlighted addresses assumptions about the entwinement of IVF embryos in the web of social, bodily, and emotional relations of the family and wider kinship group. To whom the embryo belongs is an ambiguous matter, and among a certain segment of potential embryo donors in China the opinions of family members seem to play an important role in decision-making processes:

Such a decision [to donate the embryo] must be discussed with the family as a whole, and the opinions of the others must be respected. If there is a member who disagrees, I will think about this. But it really depends on the attitude of this person. If his or her opinion is very strong, meaning opposing donation very strongly, I would not donate. I do not want to hurt the relationship between family members just because of donation. (Female IVF patient, 32 years)

Such patterns of inter-familial respect and obligations appear to be closely intertwined with culturally mediated conceptions of the human body and notions of physical interrelatedness between the generations. As one of the IVF clinicians I interviewed explained it:

You know, in Chinese cultural tradition people regard their bodies as coming from their parents, and it is seen as very precious, so we have to take good care of our bodies; we cannot give any part of it to others. So, in the Chinese tradition it is forbidden to give away … to donate your tissues or organs to others, including your cells, your gametes, which include oocytes and sperm. Therefore, [many] people cannot agree if their embryos shall be used for research.

I: What would happen if someone believes in these ideas but would still donate?

This would be an activity that means that you do not respect your parents. Your parents gave you your hair, your body, your organs, this … the whole of you. The parents gave this to you and you did not take good care of it, you gave parts of it to others. So, you don’t respect your parents.

From this perspective, donation of embryos without prior consent of the donors’ parents forms an obvious violation of culturally mediated social norms and represents a serious act of disrespect and disloyalty. This way of thinking is reflected also in a larger number of handwritten comments of survey respondents:

From a Chinese traditional point of view “we get our bodies from our parents,” so we can’t give it away casually, not to mention a new life. (Student, medicine, female, 25 years)

The traditional concepts tell us it is unsuitable to donate the embryo. I’ll give up the donation for the principle of filial piety. (Student, accountancy, male, 24 years)

## Conclusion

The findings in this article indicate once again the importance of donor-centred perspectives in bioethical debates on the use of human embryos and oocytes for research. Such research sheds light on the subjective, embodied, and emotionally charged perspectives of the women and couples who are confronted with the decision to give away parts of their bodies and on the wider socio-economic, political, and institutional contexts in which these exchanges take place. This also provides a basis for a clearer assessment of the psychological impact of donation processes and of the disruptive potential of the transfer of reproductive tissues and human genetic materials at the level of the family and wider community. Equally important, empirical evidence can play a crucial role in deconstructing politicized forms of bioethical argumentation and the often-ill-informed assumptions about “others” that inform socio-ethical debates on the impact and moral dilemmas of technology developments in the life sciences. The article has illustrated, in this regard, that Confucian-inspired ideas that a person comes into existence only at the moment of birth do not correspond to the ideas of the overwhelming majority of research participants in this study. This is of interest because this claim has played an important role in legitimizing hESC research in China and in preventing a more detailed and empirically informed exploration of public opinions. The research illustrates, furthermore, that claims cannot be upheld that ethical concerns regarding the donation and use of embryos for hESC research are something typical for Western societies but absent in China. The decision-making process for the contribution of supernumerary IVF embryos to research is, at least for the majority of participants in this study, characterized by careful normative reflections on the nature and value of these human biological resources and by an introspective assessment of the psychological and emotional consequences of the act of donation, as well as of its permissibility in the light of intergenerational patterns of obligation. While it is true that the study sample in this study is too small to derive valid conclusions at a more general level, it seems nonetheless justified to say that the Confucian-inspired notion that the life of a human being starts only at the moment of birth does not correlate with the perceptions of large numbers of Chinese IVF patients. This notion falls short in accounting for the rich plethora of meanings and needs put forward by the participants of this study.

Equally problematic appear assumptions that the value of unborn human life is generally regarded as low in China, as a result of the high number of abortions carried out in the one-child policy. Three decades of the one-child policy have clearly contributed to the devaluation of unborn forms of human life, especially at the level of state discourse and probably also within wider society. As we have shown elsewhere (Jiang and Rosemann 2018), discourses on the valuation of human embryos and fetuses differ widely across legal domains and are undergoing important changes. In patent law, ART law, and the regulation for hESC research, human gametes, embryos, and their biological originators have been defined as requiring special protection and are granted rights that prevent unauthorized removal, irresponsible use, and commodification (Jiang and Rosemann 2018). Our empirical data here suggest that a conflation of the moral positions embedded in the population policy (in which prenatal human life has been portrayed as mere “biological matter” that could be disowned and destroyed without much ethical concern) with the attitudes and perceptions of ordinary people is misleading. As the findings from our study suggest, forms of embryonic life in China are entangled in a rich web of overlapping and sometimes contradictory layers of meaning, value, emotions, and social relations, of which analysts, policymakers, researchers, and clinical staff should well be aware. While our survey data and other studies (Jin et al. 2013; Mitzkat, Haimes, and Rehmann-Sutter 2010) indicate that this is likely the case for a large proportion of the general public, this seems especially true for IVF patients, for whom embryos and gametes are of particular significance, because they embody the hope for a child after a lengthy and often painful period of infertility.

It is a significant shortcoming that bioethics discourses in China and in many other countries have for a long time approached the moral questions of embryo donation as a more abstract problem, which has been discussed in terms of broader moral and cultural categories and assumptions, rather than with regard to the specific context of IVF and the perceptions of IVF patients. As this article and others (Haimes 2008; Scully, Rehmann-Sutter, and Porz 2010) have shown, the valuation of human embryos and gametes has very particular characteristics in the IVF clinic. The context of infertility, strong hopes, and the technical process of IVF create specific forms of valuation and affection that differ from a “normal” pregnancy. These embodied and situated perceptions remain unaccounted for in the context of a hypothetical evaluation of embryo donation by non-IVF patients.

However, the findings of this study should not conceal that for approximately half of our survey respondents and IVF patients the donation of embryos for hESC seems not to constitute a problem. This is roughly the same percentage as reported from studies on embryo donation in various European countries (Haimes et al. 2008; Svendson and Koch 2008; Scully et al. 2012). As our data have shown, these persons do not expect emotional problems or ethical conflicts, and they have embraced the idea that their leftover embryos can be used for research and potentially the development of new medical treatments. It is also important to note that for many IVF patients and couples who have indicated a refusal to donate, the reasons for refusal are of a more pragmatic nature rather than fears of violating local cultural norms or the expectation of emotional or psychological conflict. The wish to keep embryos beyond the birth of a child can, for instance, also be seen as an “insurance policy” for IVF patients. As indicated by several of our interview partners, if a child becomes seriously ill and dies at an early age, there will still be spare embryos available that could be used to create another child without going through a complete IVF cycle once again, possibly even at an age when a woman’s own oocytes are no longer viable for pregnancy. A closely related point is that with the gradual relaxation of the one-child policy during the last years, many couples were literally (cyro-)banking on a change in China’s birth politics. Indeed, the transition from a one-child to a two-child policy on 1 January 2016 rendered large numbers of (frozen) embryos legally available for thousands of couples to have a second child (Wahlberg 2016). The fact that frozen IVF embryos are often seen as a sort of “reserve reproductive capital” that is valuable for IVF couples, even after their initial reproductive wish is fulfilled, is well documented in our interviews and also other studies from a US–European context (Haimes and Taylor 2009; Goswami, Murdoch, and Haimes 2015). However, the specific legal and regulatory context of China has impacted on embryo valuation and donation in a specific way. Surprisingly enough, despite their significance, many of these insights and the viewpoints and perspectives of IVF patients (as the actual donors of human embryos for research) have rarely been heard in ethical debates on hESC, neither in China nor in many other countries.

## Footnotes

1 Article 5 of patent law, People’s Republic of China, promulgated by the Standing Committee, National People’s Congress December 27, 2008. http://www.wipo.int/wipolex/en/ details,jsp?id=6511. Accessed August 1, 2015.

2 Altogether, twenty-nine students indicated having religious backgrounds: eleven Buddhist; five Christian; one Muslim; twelve other.
